# Synergistic Anticancer Activity of Cannabinoids and Terpenes Against Triple-Negative Breast Cancer Resistance

**DOI:** 10.3390/ijms27062730

**Published:** 2026-03-17

**Authors:** Mounika Aare, Jassy Mary Lazarte, Aakash Nathani, Breana Boirie, Tamiel N. Turley, John A. Copland, Mandip Singh

**Affiliations:** 1 College of Pharmacy and Pharmaceutical Sciences, Florida A&M University, Tallahassee, FL 32307, USA; mounika1.aare@famu.edu (M.A.); jassy.lazarte@famu.edu (J.M.L.); aakash1.nathani@famu.edu (A.N.); breana1.boirie@famu.edu (B.B.); 2 Cancer Biology Department, Mayo Clinic, Jacksonville, FL 32224, USA; turley.tamiel@mayo.edu (T.N.T.); copland.john@mayo.edu (J.A.C.III)

**Keywords:** cannabichromene, β-caryophyllene, combination therapy, triple-negative breast cancer

## Abstract

Triple-negative breast cancer (TNBC) remains highly aggressive and refractory to conventional treatments, underscoring the need for novel combination strategies. Here, we employed 2D and 3D in vitro models, transcriptomic profiling, and in vivo xenograft studies to evaluate the anticancer efficacy of cannabinoids combined with the terpene β-caryophyllene (BC) in resistant TNBC models. Among the tested cannabinoids, cannabichromene (CBC) exhibited the greatest potency, and its combination with BC at sub-toxic concentrations significantly reduced IC_50_ values, enhanced cytotoxicity in spheroids, and suppressed colony formation and migration. The combination treatment induced pronounced G1 cell cycle arrest and increased apoptotic cell death. Western blot analyses revealed downregulation of PARP, Survivin, mTOR, Vimentin, Glypican-5, and PD-L1, while RNA sequencing demonstrated suppression of proliferative and migratory signaling pathways alongside activation of apoptosis, autophagy, and ferroptosis-related pathways. In vivo, CBC + BC significantly inhibited tumor growth in MDA-MB-231 xenografts, outperforming single-agent treatments. Collectively, these findings demonstrate that BC synergistically enhances cannabinoid activity, yielding superior antiproliferative and anti-migratory effects, and highlight this combination as a promising therapeutic strategy for resistant TNBC.

## 1. Introduction

Conventional therapies, such as radiation, chemotherapy, surgery, immune and targeted therapies, remain among the most widely used treatments for cancer [1]. Chemotherapy is considered the standard of care in the first-line treatment of many different types of cancers, including lymphoma, leukemia, and small-cell lung cancer. Notwithstanding, it can also target healthy cells, leading to significant adverse side effects, diminishing the quality of life for patients. Despite advancements in chemotherapy over the last decade through the use of a combination of drugs and delivery systems [2,3,4], treatment failures continue to persist, attributed to drug-resistant cancer cells [5,6,7]. Drug resistance is a significant challenge for effective treatment in patients with advanced and metastatic cancers, and is a common cause of treatment failure [8]. Broadly classified as acquired or endogenous, drug resistance has been found to be associated with an increase in DNA damage repair, apoptosis suppression, target switching, drug efflux, and cell cycle checkpoint modifications [9]. The initial response to the emergence of a resistance to single-agent chemotherapy was to employ agents with distinct non-overlapping modes of action–collectively known as polychemotherapy. Combination therapy is a highly effective strategy for overcoming drug resistance in cancer treatment and can provide remarkable efficacy in treating lymphomas and hepatocellular, pancreatic, breast, and testicular malignancies [5,10,11,12]. Triple-negative breast cancer (TNBC) remains one of the most aggressive breast cancer subtypes with limited targeted treatment options and a higher prevalence among African American women. TNBC often exhibits intrinsic chemoresistance and poor prognosis; therefore, exploring novel combination strategies to enhance chemotherapy response is essential [13].

First introduced in 1998 by Mechoulam and Ben-Shabat, the endocannabinoid system, comprising endocannabinoids, CB1/CB2 receptors, and their metabolic enzymes, has been proposed to exhibit an “entourage effect”. The entourage effect describes a synergistic interaction in which a range of “inactive” metabolites and closely related molecules enhance the primary endogenous cannabinoids, anandamide and 2-arachidonoylglycerol. Mechoulam and Ben-Shabat posited this as an explanation for the observation that botanical medications frequently had higher efficacy than their isolated constituents. There is substantial evidence that Botanical synergy, referring to the combined effects of “minor cannabinoids” and Cannabis terpenoids, has stronger overall therapeutic effects than any single agent alone. Despite this evidence, single-molecule synthesis remains the predominant model rather than multi-component therapies for pharmaceutical development [14,15,16].

Cannabinoids have been extensively studied as anticancer agents [17]. Munson et al. first demonstrated the anticancer potential of cannabis-derived compounds by illustrating that tetrahydrocannabinol (THC) suppresses lung adenocarcinoma cell growth both in vitro and in mice following oral administration [18]. Several studies have reported the antiproliferative potential of phytocannabinoids, endocannabinoids, and synthetic cannabinoids in various cancers, including gliomas, breast, prostate, skin, lymphoid, and lung cancers [19,20,21,22,23]. Aside from the well-characterized cannabinoids cannabidiol (CBD) and tetrahydrocannabivarin (THCV), there are several other phytocannabinoids, including cannabichromene (CBC), cannabigerol (CBG), cannabinol (CBN), cannabinolic acid (CBNA), cannabigerolic acid (CBGA), cannabidiolic acid (CBDA), cannabidivarin (CBDV), cannabigerovarin (CBGV), cannabichromevarin (CBCV), and cannabigerolic acid (CBGA), that may have anticancer potential. Minor cannabinoids are present in smaller concentrations than THC and CBD and have combined pharmacological activity that stems in part from the binding to cannabinoid receptors, transient receptor potential cation channels, and other “off-target” receptors, which may offer advantages as therapeutic agents. Trace cannabinoids, such as THCV and CBN, bind to the CB1 receptor; however, their binding activity is notably lower than that of Δ9-THC. To date, none of the minor phytocannabinoids, other than Δ8-THC, have been shown to exhibit significant psychoactive effects in a clinical setting [24].

Previous studies have demonstrated that cannabinoids can act synergistically with conventional chemotherapeutic agents to enhance anticancer efficacy and overcome drug resistance. Several reports have shown that CBD augments the cytotoxic effects of standard-of-care therapies, including doxorubicin, cisplatin, and paclitaxel, in breast, lung, and colon cancers by promoting apoptosis, inducing oxidative stress, and suppressing prosurvival signaling pathways. Beta-caryophyllene (BC), a natural bicyclic sesquiterpene found in *Cannabis sativus*, is a selective agonist of CB2 receptors. CB2 receptors are found in abundance on immune cells, and their activation has been linked to anti-inflammatory and immunomodulatory effects. Immune modulation is crucial in cancer biology, as the immune system plays a critical role in recognizing and eliminating aberrant cells with carcinogenic potential. Studies suggest that BC may, in fact, induce apoptosis in cancer cells, promoting cell death. Cell cycle regulation is critical for normal cell growth and division. BC has been investigated for its potential to arrest the cell cycle and induce apoptosis in cancer cells, preventing uncontrolled proliferation [25,26]. BC has also been evaluated as a chemosensitizer for standard chemotherapeutic drugs and for its ability to reduce chemoresistance. Jean Legault et al. reported that the use of BC in non-cytotoxic concentrations potentially enhanced the antiproliferative effects of paclitaxel by 10-fold [27]. Moreover, BC and *β*-caryophyllene oxide were found to sensitize liver cancer cells to doxorubicin by inhibiting the expression of P-glycoprotein (P-gp) [28]. Together, these findings highlight the propensity of cannabinoids and terpenes to serve as chemosensitizers, improving therapeutic efficacy while potentially reducing the required dose and associated toxicity of standard chemotherapy agents.

Despite growing evidence that cannabinoids and terpenes exhibit anticancer and chemosensitizing properties, the combined effects of minor cannabinoids and BC in TNBC, particularly across genetically and ancestrally distinct TNBC cell models, remain largely unexplored. Furthermore, the potential of these compounds to act synergistically as chemosensitizers to overcome intrinsic chemoresistance in TNBC has not been systematically investigated.

The current study aims to evaluate the combined effects of cannabinoids and terpenes in TNBC cell lines representing different racial backgrounds, MDA-MB-231 (derived from a Caucasian patient) and MDA-MB-468 (derived from an African American patient). This comparison will provide a meaningful framework to assess whether ancestry-associated molecular differences influence chemosensitization and treatment response, thereby contributing to the understanding of racial disparities in TNBC outcomes and guiding more inclusive therapeutic development.

## 2. Results

### 2.1. Selection and Synergy Assessment Across Terpenes and Cannabinoids

Given the diverse bioactivities of terpenes, we screened a panel of individual terpenes to identify which compound most effectively reduced cell viability after a 48 h treatment in MDA-MB-468 cells (Table 1).

From this initial screening, BC was found to have the strongest cytotoxic activity among the six terpenes tested in MDA-MB-231 cells, with an IC_50_ value of 32.47 ± 0.84 µM. In contrast, bisabolol (80.50 ± 0.94 µM), α-pinene (156.53 ± 0.85 µM), α-terpineol (206.8 ± 0.83 µM), myrcene (230.6 ± 0.91 µM), and γ-terpinene (408.79 ± 0.21 µM) had considerably lower potency. Subsequent evaluation of cannabinoids revealed that CBC was the most potent compound, with IC_50_ values of 7.25 ± 0.77 µM in MDA-MB-231 and 7.13 ± 0.94 µM in MDA-MB-468 cells (Table 2). Direct comparison of single agents CBC and BC reflected by higher IC_50_ values of 32.47 ± 0.85 µM and 40.2 ± 0.76 µM in the same cell lines (Table 2).

Notably, when CBC was combined with BC at sub-toxic concentrations (IC_25_), the cytotoxic activity markedly increased, yielding IC_50_ values of 0.74 ± 0.23 µM in MDA-MB-231 and 0.57 ± 0.02 µM in MDA-MB-468 cells. This finding represented a nearly tenfold increase compared to CBC alone. A similar trend was observed in 3D spheroid models, where higher IC_50_ values were expected due to the reduced drug sensitivity of 3D tumor models. As a single agent, CBC had IC_50_ values of 20.91 ± 0.28 µM in MDA-MB-231 and 14 ± 0.85 µM in MDA-MB-468 3D cultures, while BC had IC_50_ values of 90.49 ± 0.40 µM and 83.5 ± 1.93 µM, respectively. Remarkably, compared to a single agent, the CBC + BC combination retained enhanced cytotoxicity in 3D, with IC_50_ values of 5.37 ± 0.92 µM and 5.74 ± 0.98 µM, indicating roughly a fivefold increase in potency. These findings demonstrate that BC, the most potent of the terpenes, augments the anticancer efficacy of cannabinoids, including CBC, across 2D and 3D models.

Combination index (CI) analysis was performed to evaluate the synergistic effects of cannabinoids at 0.3125 to 10 µM and BC at 17.5 µM (IC_25_) concentrations in MDA-MB-231 cells. Among the tested cannabinoids, CBC had strong synergism at lower doses (CI = 0.10–0.40) with maximal effect (E ≈ 0.91) observed from 0.625 to 10 µM. CBN demonstrated moderate synergism at intermediate doses (CI = 0.38–0.59) and a shift toward antagonism at the highest concentration (CI = 1.93). CBDA exhibited consistent synergistic interactions (CI = 0.17–0.33) with increasing efficacy (E = 0.60–0.89) across the tested range. In contrast, CBG showed mild to moderate synergy at lower concentrations (CI = 0.35–0.64) but approached antagonism at higher doses (CI > 0.9). CBDV displayed weak or no synergistic effect (CI = 0.44–1.37), indicating limited combinatorial potential under the tested conditions. Overall, CBC demonstrated the most pronounced synergistic cytotoxicity when co-administered with BC in MDA-MB-231 doxorubicin-resistant cells (Table 3). To evaluate the selectivity of these compounds, we also assessed their cytotoxicity in the non-tumorigenic breast epithelial cell line MCF 10A. Across a concentration range of 1–50 µM, CBC, BC, and their combination did not induce significant cell death, indicating that these treatments selectively target cancer cells while sparing normal breast epithelial cells.

### 2.2. Combination Treatment Suppresses Long-Term Clonogenic Survival

**Figure 1:**
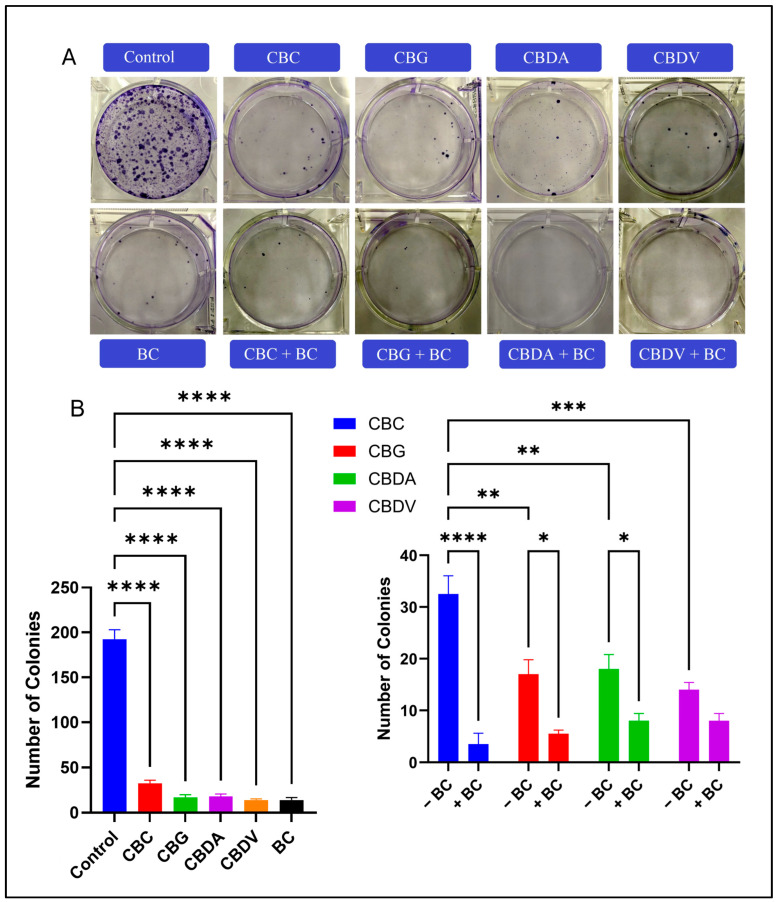
The effect of CBC, BC, and CBC + BC on colony formation in MDA-MB-231 Dox RT cells. (**A**) Representative images of crystal violet-stained colonies after treatment with individual cannabinoids (CBC, CBG, CBDA, CBDV), BC, and their respective combination treatments (CBC + BC, CBG + BC, CBDA + BC, CBDV + BC) for 14 days. The number of colonies was markedly reduced in the combination treatment groups compared to either treatment alone. (**B**) Quantitative analysis of colony numbers. The left panel displays the inhibitory effects of individual cannabinoids compared to the control. The right panel represents the synergistic reduction in colony formation when cannabinoids were combined with BC treatment. Data are presented as mean ± SD (n = 3). Statistical significance was determined using one-way ANOVA followed by Tukey’s post hoc test (* *p* < 0.05, ** *p* < 0.01, *** *p* < 0.001, **** *p* < 0.0001).

To evaluate the long-term proliferative effects, a colony formation assay was performed. The assay revealed a significant reduction in the colony-forming ability of MDA-MB-231 cells following treatment with individual cannabinoids and in combination with BC (Figure 1A). Treatment with the single agent CBC, CBG, CBDA, CBDV, and BC markedly decreased the number of colonies to 15 ± 3, 10 ± 2, 12 ± 2, 11 ± 2, and 8 ± 1, respectively. Conversely, the control group formed numerous colonies, with an average of 200 ± 10 colonies. The combination of cannabinoids and BC resulted in little to no colony formation, with nearly 0–2 visible colonies per well, indicating a greater suppression of long-term clonogenic survival compared to the single agent. Statistical analysis of clonogenic survival under single-agent and combination treatments demonstrated significant differences across all groups (*p* < 0.05 to *p* < 0.0001). The CBC + BC combination had the greatest statistical difference when compared to all other pairs (*p* < 0.0001; Figure 1B).

### 2.3. Combination Therapy Produces Distinct Cell Cycle Perturbations

**Figure 2:**
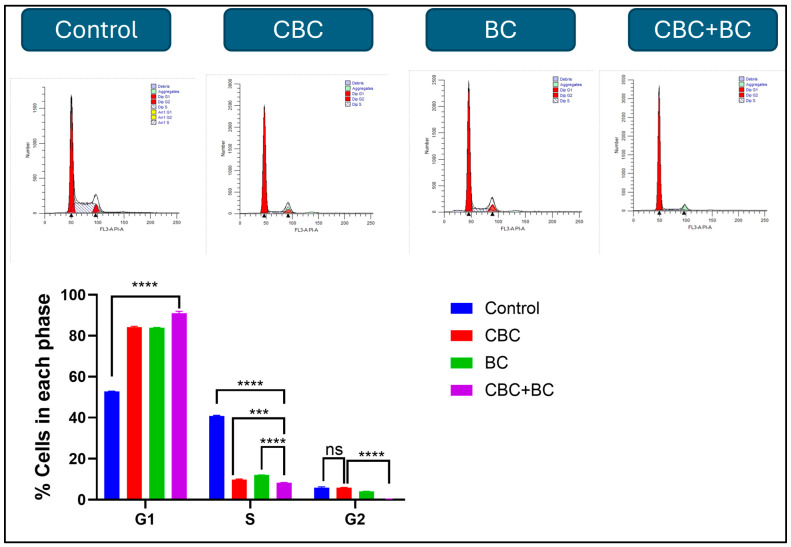
Cell cycle distribution following treatment with CBC, BC, and CBC + BC. The effects of CBC, BC, and their combination on the cell cycle in MDA-MB-231 DOX RT cells. The cells were treated for 48 h, stained with PI, and analyzed by Flow cytometry. Quantified G0/G1, S, and G2/M populations are shown as mean ± SD. The G2/M population in the CBC + BC combination is essentially absent, reflecting the strong cytotoxic effect of the treatment. Statistical significance was determined using one-way ANOVA followed by Tukey’s post hoc test (*** *p* < 0.001, **** *p* < 0.0001).

Cell cycle distribution in MDA-MB-231 cells was analyzed following treatment with CBC, BC, and in combination (CBC + BC) using flow cytometry. Significant alterations were observed in the proportion of cells across different phases compared with the control (Figure 2). In the untreated control group, 54.2 ± 2.1% of cells were in the G1 phase, 38.6 ± 1.8% in the S phase, and 7.2 ± 0.9% in the G2/M phase. CBC treatment significantly increased the G1 population to 82.4 ± 1.5% (*p* < 0.001), reduced the S phase fraction to 13.1 ± 1.3% (*p* < 0.001), and G2/M phase to 4.5 ± 0.7%. Similarly, BC treatment resulted in a G1 population of 78.3 ± 1.9% (*p* < 0.01), an S phase of 15.7 ± 1.1% (*p* < 0.01), and G2/M phase of 6.0 ± 0.8%. The CBC + BC treatment had the most pronounced G1 arrest, with 88.6 ± 1.2% of cells in G1, and a significant decrease in both S (8.9 ± 0.8%) and G2/M (2.5 ± 0.5%) populations (*p* < 0.0001 vs. control and single treatments), indicating a synergistic enhancement of G1 phase accumulation upon combination treatment.

### 2.4. Live-Cell Imaging Confirms Enhanced Cytotoxicity with Combination Therapy

**Figure 3:**
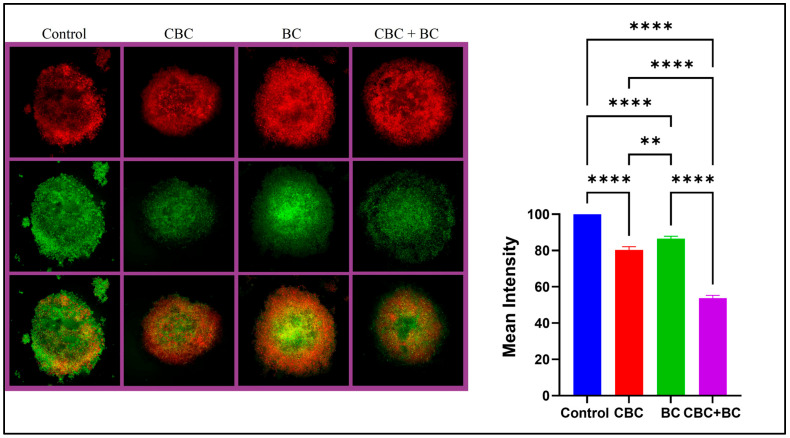
Dual acridine orange/ethidium bromide fluorescent staining of MDA-MB-231 DOX RT 3D spheroids. The cells were treated with (a) Control, (b) IC_50_ concentration of CBC, (c) IC_50_ concentration of BC, and (d) IC_50_ concentration of CBC + BC. Live cells fluoresced green (acridine orange staining), whereas dead cells fluoresced red (ethidium bromide staining). Bar graph represents relative green/red intensity (% of control). Statistical significance was determined using one-way ANOVA followed by Tukey’s post hoc test (** *p* < 0.01, **** *p* < 0.0001). Scale bar = 200 µm.

To evaluate apoptosis in MDA-MB-231 3D spheroids, a Live/Dead assay was performed following treatment with CBC, BC, and in combination. The mean fluorescence intensity was highest in control spheroids (100 ± 2.4) and was reduced following treatment with CBC (82.6 ± 3.1), BC (88.6 ± 2.7), and CBC + BC (56.2 ± 2.8), with a 44% reduction in live-cell signal, relative to the control. The differences between CBC + BC and all the other groups were statistically significant (*p* < 0.0001), signifying the greatest apoptotic response among all treatment groups. Microscopic analysis supported these findings, with the control spheroids displaying uniform green fluorescence and minimal red signal, suggesting high viability. While single-agent CBC- and BC treated spheroids exhibited moderate red fluorescence, indicating partial cell death. CBC + BC treated spheroids displayed intense red and reduced green fluorescence, reflecting enhanced apoptosis and disrupted spheroid integrity (Figure 3).

### 2.5. Combination Therapy Attenuates Cancer Cell Migration

**Figure 4:**
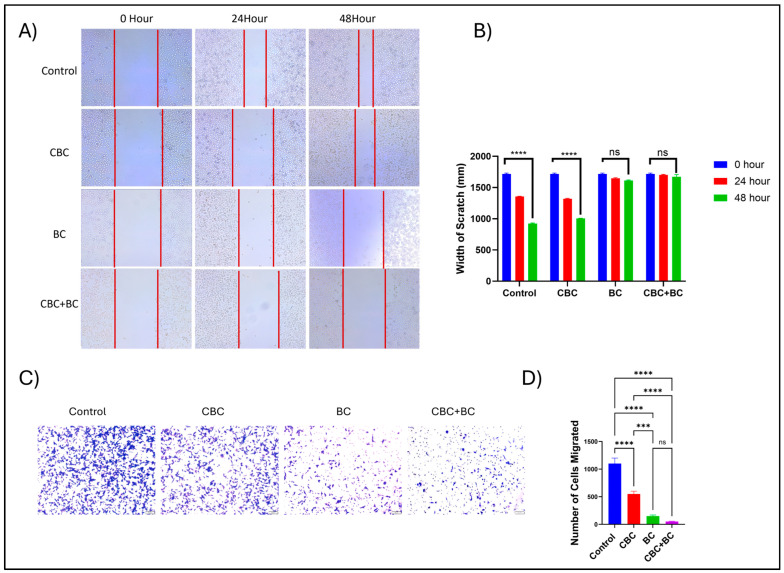
Effects of CBC, BC, and their combination on TNBC cell migration and invasion. (**A**) Insert-based wound healing assay showing the effects of CBC, BC, and their combination on cell migration. MDA-MB-231 Dox RT cells were seeded using inserts to generate a defined cell-free gap and treated with CBC, BC, and combination immediately after insert removal. Representative images were acquired at 0, 24, and 48 h. Scale bar = 100 µm. (**B**) Quantification of wound closure as the width of the scratch (mm) over time (mean ± SD, n = 3). The combination treatment significantly inhibited cell migration compared to individual treatments at 48 h. (**C**) Transwell invasion assay showing the effects of CBC, BC, and their combination on cell invasion. Cells were seeded in the upper chamber of Matrigel-coated transwells and treated with CBC, BC, or the combination. After 24 h, invaded cells were fixed, stained, and imaged. Scale bar = 100 µm. (**D**) Quantification of invaded cells (mean ± SD, n = 3) demonstrates a marked reduction in invasion with the combination treatment compared to single treatments. Statistical analyses were performed using one-way ANOVA followed by Tukey’s post hoc test (*** *p* < 0.001, **** *p* < 0.0001).

The impact of CBC and BC, as a single agent and in combination, was evaluated using a migration assay in MDA-MB-231 cells (Figure 4). In the control group, cells migrated efficiently, with a scratch width that decreased from 1780 ± 28 μM at 0 h to 694 ± 15 μm at 48 h (*p* < 0.0001). Over a 48 h period, treatment with CBC partially reduced cell migration, as the width narrowed by 1.8-fold (*p* < 0.0001). In contrast, single-agent BC and CBC + BC combination treatment produced a strong inhibitory effect on migration, by maintaining a scratch width of 1600–1700 μM across 0, 24, and 48 h, with no statistically significant changes (ns). Quantitative analysis confirmed these findings, demonstrating that while the control and CBC groups displayed significant closure of the wound area, the BC and combination groups exhibited almost complete inhibition of motility. Consistent with these observations, transwell migration analysis revealed that CBC treatment reduced cell migration by 1.8-fold relative to control (*p* < 0.01). BC treatment resulted in a 5-fold reduction in migrated cells compared to control (*p* < 0.0001). Notably, the CBC + BC combination produced the strongest inhibitory effect, leading to an 8.5-fold decrease in cell migration relative to control (*p* < 0.0001), and was significantly more effective than either CBC or BC alone (*p* < 0.05). These results suggest that BC enhances the anti-migratory effect of CBC, leading to a synergistic suppression of cancer cell migration.

### 2.6. Antitumor Activity of Combination Treatment in a Xenograft Model

**Figure 5:**
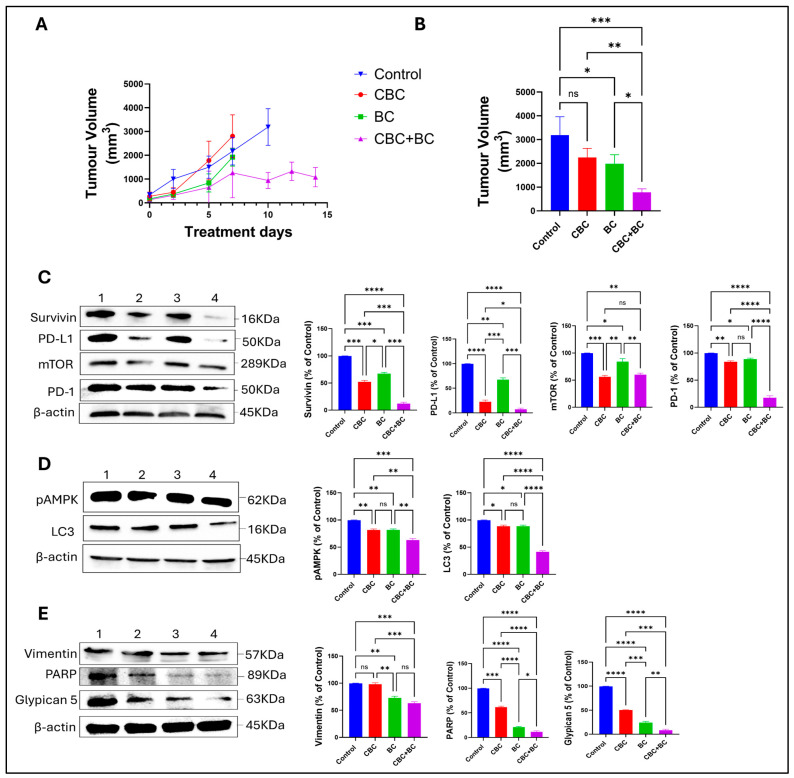
Effects of CBC, BC, and CBC + BC in an MDA-MB-231 cell-derived xenograft model. (**A**) Tumor volume progression in mice treated with vehicle control, CBC, BC, or the combination of CBC + BC. (**B**) Quantification of tumor volume at day 10 post-treatment initiation. (**C**–**E**) Representative Western blot analysis of tumor tissue lysates showing modulation of proteins associated with immune signaling (PD-1, PD-L1), apoptosis and survival (PARP, Survivin), metabolic and stress signaling (pAMPK), epithelial–mesenchymal transition and adhesion (Vimentin), and autophagy and growth signaling (LC3, mTOR) following treatment with CBC, BC, or CBC + BC. Protein expression levels were normalized to β-actin. Data are presented as mean ± SD (n = 3). Statistical significance was determined using one-way ANOVA followed by post hoc testing. * *p* < 0.05, ** *p* < 0.01, *** *p* < 0.001 and **** *p* < 0.0001 versus control.

The antitumor efficacy of CBC, BC, and in combination was evaluated in a cell-derived xenograft (CDX) mouse model of TNBC (MDA-MB-231). CBC + BC significantly reduced the mean tumor volume of 950 ± 120 mm^3^ (mean ± SD, n = 5), maintained throughout the study, with a 70% decrease when compared with the tumor volumes of control 2000 ± 180 mm^3^ by day 7 and 3100 ± 220 mm^3^ by day 14 (*p* < 0.001; Figure 5A,B). Single-agent CBC (15 mg/kg) moderately delayed tumor growth, maintaining volumes around 1200 ± 150 mm^3^ until day 5, followed by an increase to 2600 ± 200 mm^3^ at day 14 (*p* < 0.05 vs. control). BC (100 mg/kg) single-agent treatment resulted in slower tumor growth, with mean volumes of 1500 ± 140 mm^3^ on day 7 and 2300 ± 210 mm^3^ by day 14 (*p* < 0.01 vs. control; Figure 5A,B). This CDX model demonstrates an effective combination of CBC + BC that recapitulates antitumor synergist activity observed in the 2D and 3D spheroid models.

### 2.7. Differential Gene Expression Highlights Mechanisms of Therapeutic Action

**Figure 6:**
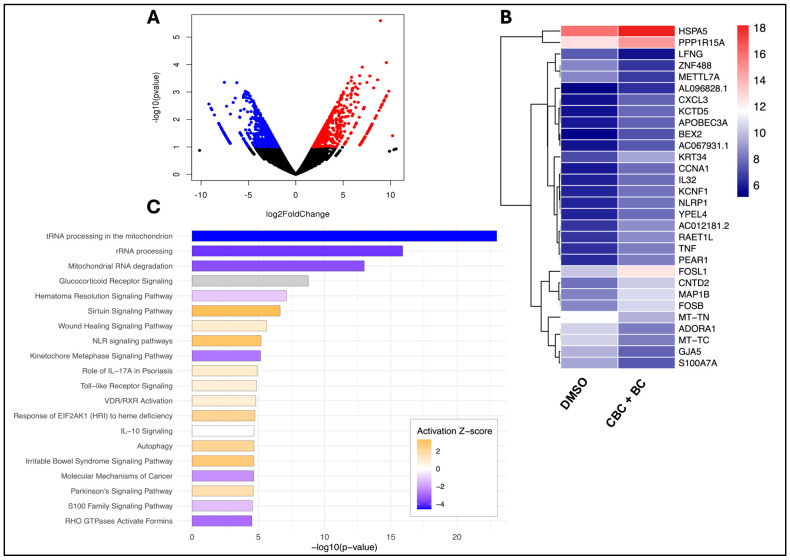
Transcriptomic profiling of CBC + BC treatment in MDA-MB-468 TNBC. (**A**) Volcano plot depicting significantly 391 upregulated (red) and 349 downregulated (blue) genes in CBC + BC-treated MDA-MB-468 cells compared with DMSO. (**B**) Heatmap of the top 30 DEGs (*p* < 0.05; log_2_ fold change ≥ 1 or ≤−1) after 24 h CBC + BC treatment (IC_50_). Genes are hierarchically clustered and color-scaled by rlog-transformed expression. (**C**) Canonical pathway enrichment analysis (IPA) highlighting key pathways altered by the combination treatment, ranked by −log_10_ (*p*-value) and colored by activation z-score.

RNA sequencing was performed to determine the transcriptional consequences of CBC + BC treatment in MDA-MB-468 TNBC cells relative to DMSO control. Differential expression analysis identified 740 significantly up- and downregulated genes involved in cell-cycle control, inflammation, and metabolic stress (|log_2_FC| ≥ 1, *p* < 0.05; Figure 6A). CBC + BC treatment elicited a strong upregulation of inflammatory mediators *CXCL3* (log_2_FC = 7.51; *p* = ${4.5\times 10}^{-4}$), *TNF* (log_2_FC = 6.00; *p* = ${7.3\times 10}^{-4}$), and *NLRP1* (log_2_FC = 6.31; *p* = ${6.5\times 10}^{-4}$), as well as genes involved in cell-cycle and signaling regulators, including *CCNA1* (log_2_FC = 7.81; *p* = ${2.6\times 10}^{-4}$), *KCNF1* (log_2_FC = 5.95; *p* = ${9.0\times 10}^{-4}$), and *YPEL4* (log_2_FC = 5.81; *p* = ${1.2\times 10}^{-3}$). Consistent with the activation of ER- and oxidative-stress pathways, the strongest responses were observed in stress-adaptive genes: *HSPA5* (log_2_FC = 5.50; *p* = ${4.8\times 10}^{-4}$) and *PPP1R15A* (log_2_FC = 5.02; *p* = ${9.9\times 10}^{-4}$). In contrast, *METTL7A* was significantly downregulated (log_2_FC = −5.43; *p* = ${1.2\times 10}^{-3}$), indicating suppression of metabolic and ER-associated functions (Figure 6B). Pathway enrichment analysis revealed a significant modulation of pathways related to mitochondrial dysfunction and RNA metabolism disruption including tRNA processing in the mitochondrion (−log_10_(*p*) = 23.0; z = −4.58), rRNA processing (−log_10_(*p*) = 15.9; z = −3.87), and mitochondrial RNA degradation (−log_10_(*p*) = 13.0; z = −3.46; Figure 6C). Several additional canonical pathways were significantly enriched, including NLR signaling pathways (−log_10_(*p*) = 5.2; z = 3.00), sirtuin signaling pathway (−log_10_(*p*) = 6.7; z = 3.30), kinetochore metaphase signaling pathway (−log_10_(*p*) = 5.2; z = −2.71) and autophagy (−log_10_(*p*) = 4.7; z = 2.18). Together, these results suggest that CBC + BC drives a coordinated transcriptional response that suppresses proliferation and pushes the cells toward stress-induced cell death.

### 2.8. Protein-Level Validation of Key Pathways Altered by Combination Treatment

**Figure 7:**
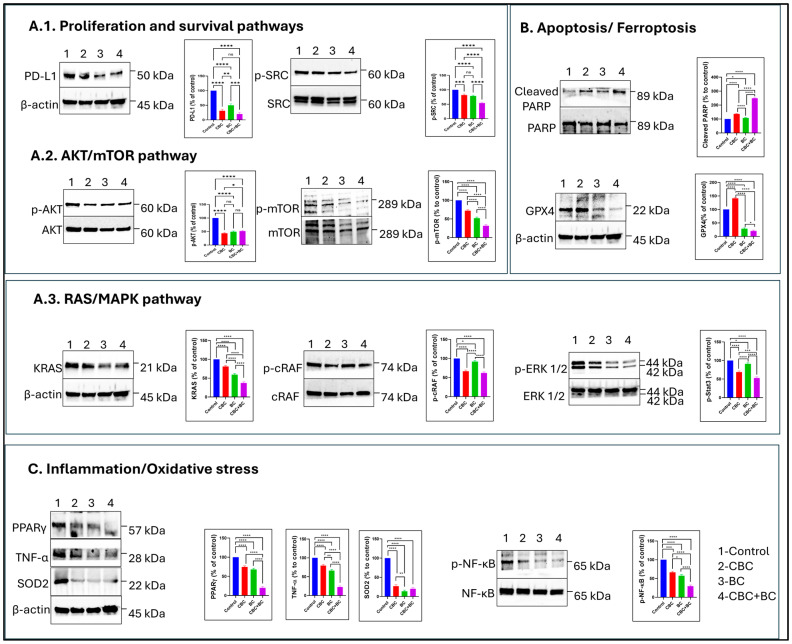
CBC and BC combination modulates survival, inflammatory, and apoptotic signaling in MDA-MB-468 cells. MDA-MB-468 cells were treated with CBC, BC, and combination (CBC + BC) for 48 h. (**A.1**–**A.3**) Western blot analysis of proliferation and survival signaling proteins, including PD-L1, p-AKT, and p-ERK1/2. (**B**) Expression of apoptotic marker cleaved PARP following single and combination treatments, as well as ferroptosis marker GPX4. (**C**) Analysis of inflammatory and oxidative stress signaling proteins, including PPARγ, TNF-α, and p-NF-κB. β-Actin was used as a loading control for all blots. Representative immunoblots are shown alongside corresponding densitometric quantification expressed as bar graphs. Data represent mean ± SD from independent experiments. Statistical significance was determined using one-way ANOVA followed by Tukey’s post hoc test (* *p* < 0.05, ** *p* < 0.01, *** *p* < 0.001, **** *p* < 0.0001). Combination treatment (CBC + BC) shows enhanced modulation of key signaling and apoptotic proteins compared to individual treatments.

To further investigate transcriptomic findings, we examined protein expression in MDA-MB-468 cells. Following treatment with CBC, BC, and in combination for 48 h, consistent with the RNA sequencing results, multiple signaling pathways related to proliferation, apoptosis, inflammation, ferroptosis, and oxidative stress were significantly altered. Western blot analysis for PD-L1 showed an appropriate decrease in PD-L1 expression, with the combination therapy achieving the lowest PD-L1 levels (~65%, *p* < 0.05; Figure 7). Moreover, phosphorylated AKT and mTOR levels were significantly reduced by 70% and 68%, respectively (*p* < 0.01), without changes in total AKT or mTOR, indicating inhibition of the AKT/mTOR axis. Similarly, phosphorylation of ERK1/2 decreased by 72% and 65% (*p* < 0.01), confirming suppression of the RAS/MAPK pathway. An increase in the cleaved PARP (3.2-fold, *p* < 0.001), with p-NF-Κb reduced by 60% (*p* < 0.05), indicated enhanced apoptotic signaling. The inflammatory markers COX-2 and phosphorylated NF-κB p65 were downregulated, with NF-κB showing an 82% reduction (*p* < 0.001). Ferroptosis-related protein GPX4 was significantly decreased (*p* < 0.01). Additionally, SOD2 expression decreased compared to control (*p* < 0.001), suggesting oxidative stress activation and antioxidant response (Figure 7).

## 3. Discussion

In this study, we demonstrated that the anticancer potential of cannabinoids can be markedly enhanced through a combination with terpenes, particularly BC, in breast cancer cell models. Synthetic forms of several minor cannabinoids and terpenes derived from *Cannabis sativa* were screened for their anticancer efficacy in the MDA-MB-231 and MDA-MB-468 cell lines. Our results demonstrate that cannabinoids exhibit similarly potent cytotoxicity, whereas BC was found to have the highest potency among the terpenes tested, albeit with a higher IC_50_ when compared to cannabinoids. This finding strongly aligns with previous studies that demonstrate the intrinsic anticancer activity of BC by inducing apoptosis and suppressing angiogenesis across multiple cancer types [25,26,29,30]. Although cannabinoids were confirmed to be more potent as single agents, terpenes, importantly, BC showed significant value as synergistic agents capable of amplifying cannabinoid efficacy. Findings that are consistent with the concept of the “entourage effect,” in which terpenes modulate cannabinoid activity and the two exert reciprocal effects.

In this study, we used pure cannabinoids and the terpene BC rather than whole plant extracts. Using isolated compounds allows precise dosing, reproducibility, and mechanistic interpretation of individual and combination effects. However, we acknowledge that whole-plant extracts contain additional minor cannabinoids, terpenes, and other bioactive constituents that could contribute to synergistic “entourage” effects. Future studies could explore complex plant matrices to evaluate potential additional benefits in more physiologically relevant contexts.

In combination, cannabinoids and BC exhibit a pronounced synergistic effect that leads to a substantial reduction in IC_50_ values across multiple resistant cancer cell lines. The combination index (CI) analysis confirmed synergism for most cannabinoid-BC combinations, except for CBN + BC, which showed additive or variable effects depending on the concentration, an observation that reflects established pharmacological principles that drug combinations may yield additive, antagonistic, or synergistic outcomes depending on the molecular interaction networks [31]. Prior studies have shown similar terpene-mediated enhancements on chemotherapy or natural product agents, in which BC amplified the activity of cisplatin in lung cancer models [32]. The magnitude of enhancement in our study strongly suggests that BC can potentiate cannabinoid-mediated anticancer activity beyond what has been previously documented in the literature.

Consistent with earlier reports that cannabinoids and terpenes can suppress cancer cell proliferation, migration, and metastasis [33], our colony formation assays demonstrated that treatment with CBC, CBG, CBDA, and CBDV markedly inhibited clonogenic survival of MDA-MB-231 DOX-resistant cells. Furthermore, the additional reduction observed in the combination groups suggests a synergistic interaction between cannabinoids and BC. Prior work has demonstrated that cannabinoids modulate key cell-survival and proliferative signaling pathways-ERK, AKT, mTOR, and caspase activation-thereby reducing long-term survival of cancer cells. The enhanced suppression of colony formation with the combination treatment likely results from BC’s ability to engage CB2 and PPAR-γ receptor activity and further modulate survival signaling, thereby amplifying the antiproliferative and pro-apoptotic signaling induced by cannabinoids [34,35].

Cell cycle analysis revealed that single-agent CBC and BC treatments induced G1-phase arrest in MDA-MB-231 cells, whereas the CBC + BC combination elicited the highest G1 accumulation, with the greatest reduction in S-phase and G2/M populations. These results suggest an effective inhibition of the G1–S transition and a strong antiproliferative response. Prior work has described a cannabinoid-induced G0/G1 arrest-via down-regulation of cyclin D1, CDK4/6, and E/E2 expression-in colorectal and colon carcinoma models [36,37]. The robust G1 arrest observed with our combination may stem from additive or synergistic interference with CDK/cyclin control, DNA-synthesis initiation, and checkpoint regulation (p21, p27 up-regulation). The observed cell-cycle blockade dovetails with the apoptosis induction and supports the mechanistic narrative of impaired proliferation followed by programmed cell death.

Extending these observations to further elucidate the functional consequences of the combination treatment, Live/Dead assays revealed that the combination of CBC + BC induced the strongest apoptotic activity, reflected by an increase in red fluorescence in treated spheroids and a substantial reduction in viability relative to single-agent treatments. The morphological disruption of spheroid integrity in the combination group underscores the depth of the effect in 3D tumor-mimic structures. These functional data align well with the Western blot findings, which show elevated PARP cleavage and down-regulation of Survivin. Mechanistically, a growing body of research supports the idea that cannabinoids induce apoptosis through mitochondrial pathway activation, caspase cleavage, and modulation of the Bcl-2 family. Notwithstanding, terpenes, including BC, induce apoptosis via ROS induction, mitochondrial depolarization, and caspase-3 activation [38,39,40]. The ability to converge apoptotic and oxidative stress pathways, by combination therapy, thus offers a mechanistic explanation for enhanced cytotoxicity.

In agreement with our wound-healing data, the CBC + BC combination treatment markedly inhibited cell migration compared to either agent alone. While each component individually slowed migration, their co-treatment nearly prevented wound closure, implicating a cooperative mechanism of action. The observed suppression is in accord with previous findings in which cannabinoids reduce migration and invasion by interfering with cytoskeletal re-organization, focal adhesion kinase (FAK), and matrix metalloproteinases (MMPs) [41]. Terpenes such as BC have similarly been shown to inhibit epithelial–mesenchymal transition (EMT) and angiogenesis via multiple pathways (including MAPK/STAT3 and integrin signaling) in cancer models [42,43]. Consistent with these observations, transwell migration assays provided independent validation of the anti-migratory effects of CBC and BC. While CBC alone exerted a modest inhibitory effect, BC markedly impaired migratory capacity, and the combination treatment produced the greatest suppression of cell migration. Together, these results suggest that the CBC + BC combination effectively impairs both migratory and proliferative capacities of resistant breast cancer cells, highlighting their potential to limit tumor spread and recurrence.

To validate our findings in a preclinical context, an MDA-MB-231 xenograft study was conducted. In vivo, the CBC + BC combination treatment significantly suppressed tumor growth compared with individual treatments, confirming that the in vitro mechanistic insights translate to measurable antitumor response. Analysis of protein expression in tumor samples further substantiated the mechanistic findings of the down-regulation of proliferation-associated proteins (mTOR, Survivin, pAMPK), migratory markers (Vimentin, Integrin, Glypican-5), and immune-evasion checkpoints (PD-L1, PD-1). Interestingly, BC alone had a stronger effect on migratory proteins (Vimentin, Glypican-5) than CBC alone, consistent with BC’s known anti-migratory/anti-metastatic capacity [44]. The marked down-regulation of PD-L1 in the combination group suggests that the CBC + BC treatment may not only inhibit tumor growth and motility but also modulate immune-suppression mechanisms, an emerging field of interest in cannabinoid research. These results underscore the hypothesis that CBC + BC combinations can disrupt tumor survival networks, motility/invasion circuitry, and tumor-immune interactions, offering a multi-pronged anticancer strategy.

To extend mechanistic understanding beyond phenotypic assays, we performed RNA-sequencing on treated and untreated MDA-MB-468 cells, a genetically distinct, African American-derived TNBC line under-represented in cannabinoid research. The transcriptomic profiling of CBC + BC treatment revealed a strong upregulation of inflammatory (TNF, CXCL3) and stress-adaptive genes (HSPA5, PPP1R15A), as well as downregulation of metabolic regulators (METTL7A). These DEGs aligned with canonical pathways predictive of the inhibition of mitochondrial RNA processing, kinetochore/metaphase signaling (suppressed proliferation), and activation of innate immune and stress-response pathways (NLR and sirtuin signaling, autophagy).

The protein level analysis supported these pathway predictions by a significant attenuation of PI3K/AKT and MAPK signaling by the inhibition of AKT, ERK1/2, MEK, and mTOR phosphorylation. These changes reflect a coordinated reprogramming of cellular survival, motility, and death mechanisms. The findings align with previous reports that cannabinoids can trigger autophagy and ferroptosis (for example, via GPX4 down-regulation) in aggressive cancer subtypes [45,46]. BC has been shown to regulate intracellular antioxidant systems, increase ROS, and induce ferroptosis in certain cancers [47,48]. The suppression of metabolic and mitochondrial RNA-processing pathways and mTOR signaling in the RNA-seq dataset suggests that CBC + BC promotes metabolic stress and vulnerability, contributing to cell death. Moreover, suppression of integrin, wound-healing, and motility-associated pathways aligns with the impaired motility observed in the migration assays. The consistency of transcriptomic trends in MDA-MB-468 suggests that the CBC + BC synergy reflects a broad-spectrum mechanism across TNBC subtypes rather than a single-cell-line idiosyncrasy.

**Figure 8:**
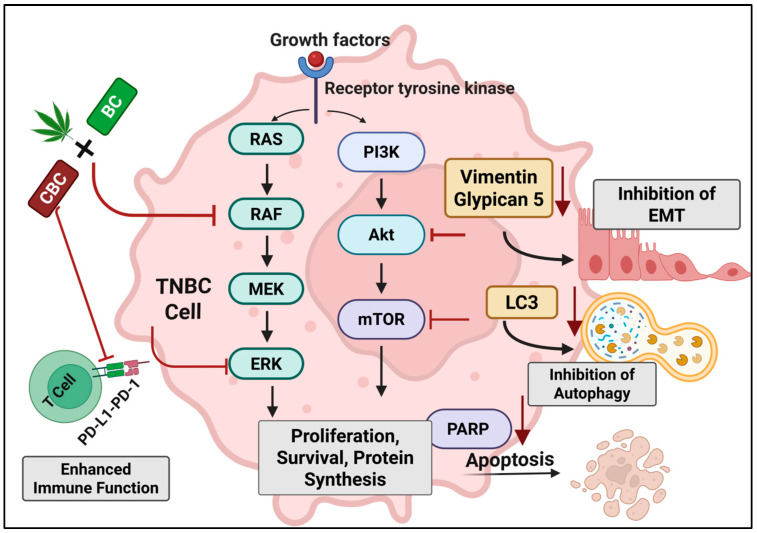
Schematic representation of the proposed molecular mechanisms underlying the anticancer activity of the CBC + BC combination in TNBC cells. CBC and BC act synergistically to inhibit key oncogenic signaling pathways, including RAS–RAF–MEK–ERK and PI3K–Akt–mTOR, leading to reduced proliferation, survival, and protein synthesis. The combination also downregulates EMT markers (Vimentin, Glypican-5), resulting in inhibition of epithelial–mesenchymal transition, and suppresses autophagy via LC3 inhibition. Additionally, CBC + BC promotes apoptosis through PARP activation and enhances anti-tumor immune response by modulating PD-L1/PD-1 signaling. Overall, the combined action of CBC and BC orchestrates multiple molecular events that converge to reduce tumor growth and invasiveness.

Mechanistically, the synergy between CBC and BC likely arises from several interacting dimensions. First, receptor and signaling complementarity: CBC has been shown to act upon CB_2_, TRPV1, and TRPA1 channels (though with relatively modest affinity), whereas BC is a selective CB_2_ agonist and PPAR-γ modulator with the capacity to influence lipid membrane permeability and drug uptake [49,50]. By engaging distinct but overlapping receptor systems, the combination may elicit broader and deeper downstream signaling disruption. Second, metabolic, and redox-driven stress: BC’s ability to dysregulate antioxidant enzyme systems and generate ROS, combined with cannabinoid-driven suppression of survival signaling, creates a convergence of apoptotic, autophagic, and ferroptotic stress that resistant cancer cells may find difficult to counter-regulate [45,51]. Third, the suppression of motility and immune-evasion pathways: the combination therapy targets migration/invasion via integrins/EMT (Vimentin, Glypican-5) and immune checkpoint expression (PD-L1/PD-1), thereby attacking both tumor intrinsic and microenvironmental support mechanisms. In summary, the CBC + BC combination appears to disable tumor growth, dissemination, and immune escape simultaneously (Figure 8).

Beyond mechanistic insight, the nearly ten-fold increase in potency with the CBC + BC combination therapy highlights the possibility of treatment at a lower dose for each agent while preserving efficacy. This would have significant translational implications, potentially reducing toxicity and improving therapeutic index. Moreover, the breadth of mechanistic targeting spanning apoptosis, ferroptosis, cell-cycle arrest, motility suppression, and immune checkpoint modulation makes the combination appealing for aggressive, treatment-resistant TNBC, where monotherapy often fails due to a redundancy in survival pathways. Importantly, both CBC and BC are non-psychoactive (unlike Δ^9^-THC), which greatly enhances translational feasibility and patient acceptability. The immune-checkpoint modulation observed invites future combinations with immunotherapies, especially given emerging interest in cannabinoids as modulators of tumor-immune interactions. Lastly, the robust in vivo antitumor response supports the feasibility of moving from in vitro mechanistic studies to pre-clinical therapeutic development.

To capture complementary aspects of treatment response, this study utilized two TNBC cell lines representing distinct patient backgrounds: MDA-MB-231, derived from a Caucasian individual and MDA-MB-468 from an African American individual. Although the most extensive phenotypic characterization was performed using the cell line MDA-MB-231, this provided a comprehensive framework to highlight the effects of treatment across multiple biological levels. While transcriptomic profiling was performed only in the MDA-MB-468 cell line and in vivo studies were conducted only in MDA-MB-231, the treatment responses in both models aligned with the functional effects observed across 2D and 3D assays. The in vivo MDA-MB-231 CDX model utilized immunodeficient mice, although immune-evasion markers (PD-L1/PD-1) were suppressed. Accordingly, the effect on immune cell infiltration or activation remains to be determined in immune-competent or humanized models. Further, pharmacokinetic, biodistribution, and toxicity profiling of CBC + BC combinations must be explored to support translation. Finally, resistance mechanisms (e.g., efflux transporters, tumor heterogeneity) remain to be evaluated in the context of the combination therapy. While the present study focused on differential gene expression and canonical pathway enrichment to elucidate the transcriptional effects of CBC + BC treatment, additional pathway-level analyses may further refine mechanistic interpretation. In particular, Hallmark gene set enrichment analysis (GSEA) could provide a complementary, higher-order view of coordinated biological programs by reducing redundancy across pathway annotations and highlighting core cellular processes such as inflammatory signaling, stress responses, metabolic dysregulation, and cell-cycle control. Incorporation of Hallmark pathway analysis in future studies may therefore strengthen the integrative understanding of the transcriptional networks underlying the therapeutic response to the CBC + BC combination.

## 4. Materials and Methods

### 4.1. Materials

MDA-MB-231 and MDA-MB-468 were purchased from American Type Culture Collection (Rockville, MD, USA) and cultured in DMEM-F12 (Genesee Scientific, Morrisville, NC, USA) supplemented with 10% Fetal Bovine Serum (Biotechne, Minneapolis, MN, USA) and 1% penicillin/streptomycin at 37 °C with 5% CO_2_. Doxorubicin-resistant MDA-MB-231 cells were generated by continuous exposure of parental wild-type cells to gradually increasing concentrations of doxorubicin (0.1–25 μM) over 8–10 months, as previously described [52]. Resistance was confirmed using 2D cell viability assays, in which the IC_50_ for doxorubicin increased from 2.26 ± 0.21 μM in parental cells to 31.00 ± 1.63 μM in DOX-RT cells, corresponding to an approximately 12–14-fold increase in resistance. Resistance was further validated in 3D spheroid cultures, where DOX-RT cells exhibited a significantly higher IC_50_ value (194.61 ± 20.64 μM) [52]. Cannabinoids and terpenes were generous gifts from Open Book Extracts (Roxboro, NC, USA). Primary and secondary antibodies used in the Western blotting were procured from Cell Signaling Technology (Danvers, MA, USA). All other supplies and reagents were purchased from Sigma-Aldrich (St. Louis, MO, USA).

### 4.2. Cell Viability Studies in 2D and 3D Cultures

The anticancer potential of several cannabinoids (CBC, CBG, CBN, CBDA, CBDV) and terpenes (BC, bisabolol, myrcene, linalool, limonene, geraniol, nerolidol, α-pinene, α-terpineol, γ-terpinene) was studied against the chemo-resistant MDA-MB-231 cell line in 2D cultures. Briefly, cells were seeded in a 96-well plate at a density of 7000 cells/well. After 24 h, the cells were treated with cannabinoids and terpenes for 48 h, in a concentration-dependent manner. After 48 h, the media were replaced with MTT solution (0.5 mg/mL). Absorbance was measured using Tecan Infinite 200 PRO M Plex multimode microplate reader (Tecan, Männedorf, Switzerland) at 570 nm [53]. The combination of drug treatments was analyzed by CompuSyn software (version 1.0) to identify combination index values that define synergism (CI < 1), an additive effect (CI = 1), or antagonism (CI > 1). To validate the findings in MDA-MB-231 cells, the same methods listed above were followed with MDA-MB-468 using all cannabinoids (CBC, CBG, CBN, CBDA, CBDV) alone and in combination with BC.

Compound cytotoxicity was also assessed in 3D spheroids. Both cell lines were tagged with a nanoshuttle solution (10 μL for 10,000 cells; Greiner-Frickenhausen, Germany) by centrifugation at 800 RPM for 7 min three times. Cells were seeded in cell-repellent-surfaced 96-well plates at a density of 15,000 cells/well and kept on a magnetic drive, which aids in the formation of spheroids. Spheroids were allowed to grow for 5 days with an intermediate media change on the 3rd day. After day 5, cells were treated with single-agent cannabinoids and terpenes, and in combination in a concentration-dependent manner for 48 h. After 48 h, the media were replaced with 0.5 mg/mL MTT solution (100 μL/well) and absorbance was read at 570 nm [54,55]. To further assess selectivity, cytotoxicity was also evaluated in the non-tumorigenic breast epithelial cell line MCF 10A, across a concentration range of 1–50 µM.

### 4.3. Colony Formation Assay

MDA-MB-231 cells were seeded at a density of 500 cells/well. After 24 h, the cells were treated with cannabinoids, BC, and in combination. Cells were allowed to multiply for two weeks, with a media change twice weekly. After two weeks, cells were fixed using 4% paraformaldehyde overnight at 37 °C and stained with a 0.1% crystal violet solution for 30 min. Following the incubation period, the staining solution was removed, cells were washed thoroughly with phosphate-buffered saline (PBS) and the number of colonies was counted under a microscope [56].

### 4.4. Cell Cycle Analysis

The Sony SH800 Cell Sorter (San Jose, CA, USA) was used to assess the effect of the compounds on the cells. MDA-MB-231 cells were seeded in a 6-well plate at a density of 0.5 million cells/well. After 24 h, the cells were treated with CBC, BC, and CBC + BC at IC_25_ and IC_40_ concentrations. After 48 h of treatment, the media was aspirated, the cells were washed three times with PBS, and trypsinized. The pellet obtained post-trypsinization was fixed using 70% ethanol at 4 °C overnight. After fixation, the cells were centrifuged at 300× *g* for 10 min, and the pellet was washed with PBS by centrifugation under the same conditions as previously mentioned. Cells were then incubated with 50 μg/mL RNase A and 100 μg/mL of propidium iodide for 30 min under dark conditions at room temperature. Cell Sorter Software version 2.1.6 was used for the acquisition of the data, and the results were displayed as histograms (FL3-A vs. counts). The percentage of cells in each phase was determined by using ModFit LT 5.0 Software (Verity Software House Inc, Topsham, ME, USA) [57].

### 4.5. Live/Dead Assay

In the current study, we assessed the apoptotic potential of BC, cannabinoid single-agents, and in combination on MDA-MD-231 using the acridine orange-ethidium bromide (AO/EB) dual staining procedure. 3D spheroids were cultured using a magnetic nanoshuttle system as previously described for 5 days. On day 5, the spheroids were treated with CBC, BC, CBC + BC at IC_25_ concentrations and incubated for 48 h. After 48 h, the spheroids were treated with acridine orange (10 μL of 100 µg/mL diluted to 100 μL with PBS) and ethidium bromide (10 μL of 100 µg/mL diluted to 100 μL with PBS) and incubated at room temperature for 30 min. After incubation, the spheroids were washed thrice with PBS, and fluorescence was measured using an Olympus IX 73 fluorescence microscope (Olympus, Center Valley, PA, USA) [58].

### 4.6. Migration and Invasion Assay

Cell migration was assessed using an insert-based wound healing assay. MDA-MB-231 cells were seeded into culture inserts placed in 6-well plates and allowed to reach confluence under standard culture conditions. After cell attachment, the inserts were carefully removed to generate a defined cell-free gap. Cells were treated then with CBC, BC, and CBC + BC combinations at an IC_25_ concentration. After 48 h of treatment, scratch images were obtained, and the scratch width was measured over time using the Olympus IX 73 fluorescence microscope (Olympus, Center Valley, PA, USA) [59]. Cell migration was further evaluated using a transwell migration assay. Briefly, MDA-MB-231 DOX RT cells were serum-starved overnight, harvested, and seeded into the upper chambers of transwell inserts with porous membranes (8-μm pore size) in serum-free medium. Medium-containing serum was added to the lower chamber as a chemoattractant. Cells were treated with CBC, BC, and their combination at the time of seeding. After incubation for 24 h, non-migrated cells on the upper surface of the membrane were removed using a cotton swab. Migrated cells on the lower surface were fixed with paraformaldehyde, stained with crystal violet, and imaged using the Olympus IX 73 fluorescence microscope (Olympus, Center Valley, PA, USA). Migratory cells were quantified by using ImageJ (ImageJ 1.36; Wayne Rasband, National Institutes of Health, Bethesda, MD, USA).

### 4.7. RNA Sequencing and Pathway Analysis

Total RNA was extracted from lysates prepared from cultured MDA-MB-468 cells treated with a combination of CBC + BC at their respective concentrations alongside a DMSO control for 24 h Cell lysis, total RNA extraction, rRNA depletion, and strand-specific library preparations were collected and sequenced on the Illumina NovaSeq 6000 platform (2 × 150 bp paired-end reads; GENEWIZ from Azenta Life Sciences, South Plainfield, NJ, USA). All RNA sequencing passed standard quality control metrics for coverage. Normalized expression files (rLog-transformed matrices) and differential expression results generated by GENEWIZ were used for downstream bioinformatic analyses. Differentially expressed genes (DEGs) were filtered using a *p*-value < 0.05 and an absolute log_2_ fold change ≥ 1. DEGs were analyzed using Ingenuity Pathway Analysis (IPA; Qiagen, Redwood City, CA, USA). Canonical Pathway Analysis was performed using the Core Analysis module, which computes pathway enrichment (−log_10_ *p*-value) and activation z-scores based on directionally regulated genes. Filtered DEGs and IPA output tables were downloaded and imported into R (v4.5.2; R Foundation for Statistical Computing, Vienna, Austria). Statistical analyses and visualizations were conducted in R using ComplexHeatmap (v2.18), ggplot2 (v3.4), and tidyverse (v2.0.0) packages. Principal component analysis was not performed because only one sample per condition was tested. Transcriptomic comparisons instead used differential expressions and pathway enrichment analyses.

### 4.8. In Vivo Animal Studies

BALB/c athymic nude mice (male, 6 weeks old, Foxn1^nu^) were purchased from Envigo (Indianapolis, IN, USA) and housed under stringent pathogen-free conditions in an American Association for Accreditation of Laboratory Animal Care-approved facility. The animals were kept in typical housing cages with access to food and water, at room temperature of 37 °C and a relative humidity of 60%. Institutional Animal Care and Use Committee (IACUC) regulations of Florida A&M University were followed throughout the study (Protocol Number for animal studies: 023-02). Mice were acclimatized for a week before inducing the tumors.

MDA-MB-231 cells were mixed with VitroGel (The Well Biosciences, Monmouth Junction, NJ, USA) in a 1:1 ratio to form a suspension that was kept at 37 °C for 25 min, followed by 5 min on ice. Subsequently, 5 million MDA-MB-231 cells were injected subcutaneously into the right flank of athymic nude mice. Once tumors reached 180–200 mm^3^, animals were randomly assigned to four treatment groups (n = 6 per group). Active treatment groups were administered CBC (15 mg/kg, i.p.), BC (100 mg/kg, i.p.), or CBC + BC thrice a week for two weeks. The tumor dimensions were measured using vernier calipers throughout the 14-day treatment, and tumor volumes were calculated using the tumor volume formula: 1/2 xy^2^, where ‘x’ and ‘y’ represent the length and width of the tumors. Tumors were collected at the endpoint and processed for downstream analyses [52].

### 4.9. Western Blot Analysis

Following the protocol described by Zhang [60], MDA-MB-468 and MDA-MB-231 cells were lysed using a mixture of T-PER^TM^ protein extraction reagent (#78510; Thermo Scientific, Waltham, MA, USA), protease (#P8340), and phosphatase inhibitors (#P2850; 1:100; Sigma Aldrich, St. Louis, MO, USA). The protein content was estimated by a BCA assay. An equivalent volume of 40 µg of protein was loaded onto an SDS PAGE gel and run for protein separation. The proteins were transferred from the gels to nitrocellulose membranes, followed by blocking with 5% BSA. Blocked membranes were incubated with primary antibodies at 4 °C overnight, and secondary antibodies corresponding to the primary antibodies were added and incubated for 2 h at room temperature. Following secondary antibody removal, the blots were washed thrice with TBST and developed with ECL substrate using the ChemiDoc^TM^ XRS+ Imaging system (Bio-Rad, Hercules, CA, USA). The relative band densities were quantified using densitometric analysis software (ImageJ 1.36; Wayne Rasband, National Institutes of Health, Bethesda, MD, USA).

### 4.10. Statistical Analysis

All experiments were performed in triplicate unless otherwise stated, and data are presented as mean ± standard deviation (SD). Statistical comparisons between multiple groups were performed using one-way analysis of variance (ANOVA) followed by Tukey’s post hoc test to assess pairwise differences. A two-tailed unpaired Student’s *t*-test was used for comparisons between two groups when applicable. Statistical significance was defined as *p* < 0.05. Differential expression statistics (DESeq2 (version number 1.50.2.)) and pathway enrichment calculations (IPA Fisher’s exact test and activation z-score) were applied as described in the RNA sequencing and pathway analysis section. All other statistical analyses were conducted using GraphPad Prism (version 8.0, GraphPad Software, San Diego, CA, USA).

## 5. Conclusions

Our findings indicate that BC significantly enhances the anticancer effects of cannabinoids, particularly in resistant TNBC cells. BC not only inhibits cell migration, indicating a potential to limit metastatic behavior, but also promotes apoptosis, likely through modulation of PARP activity and suppression of anti-apoptotic proteins, thereby amplifying cell death signaling. When combined with cannabinoids, BC produces markedly stronger cytotoxic effects, resulting in pronounced G1-phase cell cycle arrest, enhanced apoptosis, and disruption of spheroid integrity compared to cannabinoids alone. These observations suggest that BC functions as a natural sensitizer, simultaneously amplifying cannabinoid-mediated cytotoxicity and targeting multiple oncogenic pathways. Collectively, these results establish a mechanistic and translational rationale for CBC + BC combination therapy, demonstrating synergistic disruption of proliferation, migration, survival signaling, metabolic adaptation, and immune-evasion networks in resistant TNBC. The integration of RNA-sequencing in MDA-MB-468 cells and mechanistic validation in MDA-MB-468 and MDA-MB-231 cells highlights both the broad applicability and translational potential of this combination therapy, supporting its development as a novel approach to overcome resistance and limit tumor progression in aggressive breast cancers.

## Figures and Tables

**Table 1:** Half-maximal inhibitory concentrations (IC_50_) values of terpene compounds in MDA-MB-2312D culture.

Compound	IC_50_ Value (µM)
β-Caryophyllene	32.47 ± 0.84
Bisabolol	80.50 ± 0.94
Alpha-Pinene	156.53 ± 0.85
Alpha-Terpineol	206.8 ± 0.83
Myrcene	230.6 ± 0.91
Gamma-Terpinene	408.79 ± 0.21

IC_50_ values represent mean ± SD from independent experiments.

**Table 2:** In vitro screening of cannabinoids, terpenes and combination treatments in TNBC cell lines in 2D and 3D cultures.

Sample Number	Compound	MDA-MB-231 Cells		MDA-MB-468 Cells	
		2D (IC_50_)	3D (IC_50_)	2D (IC_50_)	3D (IC_50_)
1	CBC	7.25 ± 0.77	20.91 ± 0.28	7.13 ± 0.94	14 ± 0.85
2	CBG	9 ± 0.84	28.62 ± 0.38	8.84 ± 0.59	20.42 ± 0.85
3	CBDA	9.3 ± 0.35	45.83 ± 0.52	9.86 ± 0.42	42.5 ± 0.85
4	CBDV	8.08 ± 0.95	32.45 ± 0.22	9.36 ± 0.22	35.5 ± 0.62
5	CBN	11.9 ± 0.92	35.29 ± 0.51	11.4 ± 0.68	32.56 ± 0.61
6	BC	32.47 ± 0.85	90.49 ± 0.40	40.2 ± 0.76	83.5 ± 1.93
7	CBC + BC (IC_25_)	0.74 ± 0.23	5.37 ± 0.92	0.57 ± 0.02	5.74 ± 0.98
8	CBG + BC (IC_25_)	1.34 ± 0.05	8.28 ± 0.81	1.04 ± 0.05	8.39 ± 0.53
9	CBDA + BC (IC_25_)	0.8 ± 0.25	7.24 ± 0.74	1.35 ± 0.07	6.91 ± 0.84
10	CBDV + BC (IC_25_)	1.14 ± 0.28	6.84 ± 0.59	2.24 ± 0.14	6.78 ± 0.44

IC_50_ values represent mean ± SD (n = 4). BC: β-caryophyllene; CBC: cannabichromene; CBG: cannabigerol; CBDA: cannabidiolic acid; CBDV: cannabidivarin.

**Table 3:** Combination index (CI) and dose–response effects of β-caryophyllene in combination with cannabinoids in MDA-MB-231 cells.

Cannabinoid (μM)	BC (μM)	CBC		CBN		CBDA		CBG		CBDV	
		Effect	CI	Effect	CI	Effect	CI	Effect	CI	Effect	CI
0.3125	17.5	0.528	0.401	0.250	0.957	0.600	0.335	0.447	0.509	0.17	1.374
0.625	17.5	0.898	0.102	0.520	0.499	0.644	0.318	0.537	0.425	0.36	0.723
1.25	17.5	0.910	0.102	0.667	0.383	0.714	0.283	0.640	0.349	0.56	0.446
2.5	17.5	0.910	0.119	0.676	0.467	0.777	0.263	0.522	0.637	0.604	0.474
5.0	17.5	0.910	0.154	0.700	0.592	0.881	0.176	0.511	0.932	0.669	0.500
10.0	17.5	0.941	0.156	0.539	1.927	0.885	0.239	0.492	1.588	0.644	0.838

## Data Availability

The original contributions presented in this study are included in the article. Further inquiries can be directed to the corresponding authors.

## Abbreviations

BC	β-Caryophyllene
CBC	Cannabichromene
TNBC	Triple-Negative Breast Cancer
